# Two decades, a new editorial stylistic feature

**DOI:** 10.1186/s10194-020-1076-y

**Published:** 2020-01-24

**Authors:** Paolo Martelletti, on behalf of all Editors and Advisory Boards

**Affiliations:** Department of Clinical and Molecular Medicine, Sapienza University of Rome, Roma, Italy

**Figure 1:**
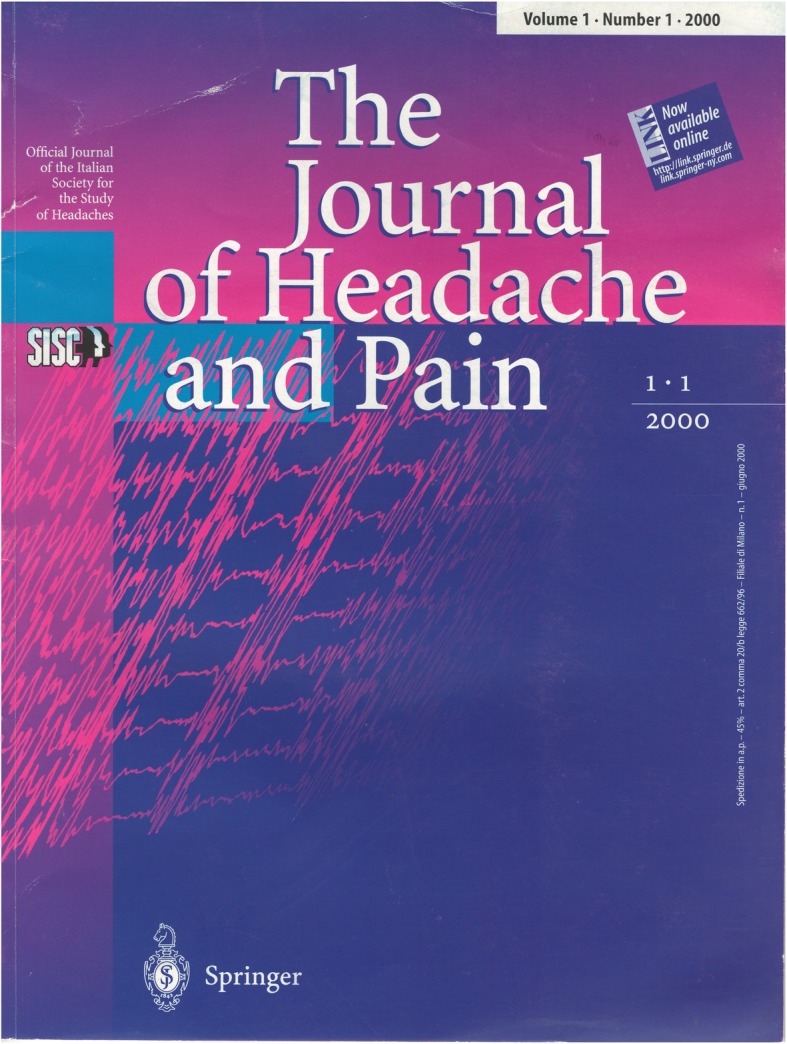
Fig. 1

Twenty years have passed since the first number of The Journal of Headache and Pain (Fig. 1). I can perfectly recall its ideation phase, rooted on the necessity of a third cultural pole in the scientific area of headaches, its organizational phase in collaboration with a giant in the editorial world, the implementation and launch phase, crowded with whispered political criticisms or with ill-concealed obstructing indifference [1].

With a continuous daily teamworking we have waited until indexing services noticed us, we dared to choose the open access option when no one would have put a penny on it, we continued our run by continuously introducing innovative initiatives and procedures. In one word, we unceasingly tested our limits.

Apart from the progressive, constant and therefore solid growth of the Impact Factor, I particularly care about how our readers have rewarded the Journal with about 1 million papers downloaded in 2019, and the increasingly permeating social activity.

We savor a moment of joy for this birthday together with the many, many scientists who over time have opted to publish their scientific works here, have shared the activities of the Boards of the Journal and *ça va sans dire* together with SpringerNature, that has made available, always and in preview, its immense technological know-how and, certainly not least, together with the affiliated Scientific Societies, the European Headache Federation and Lifting The Burden, who have given us the privilege of hosting their best publications. A special thank to the reviewers, the real engine of the Journal, who have been able to add speed to the quality of the reports.

Lastly, we will keep thinking The Journal of Headache and Pain as a privileged cradle for new ideas, other than the Thematic Series, Greppi Awards, EHF-School of Advanced Studies reviews, European Migraine Registry reports.

Thanks to all of you we have built a new stylistic feature within our scientific community.
